# Transparent ferromagnetic and semiconducting behavior in Fe-Dy-Tb based amorphous oxide films

**DOI:** 10.1038/srep27869

**Published:** 2016-06-14

**Authors:** H. Taz, T. Sakthivel, N. K. Yamoah, C. Carr, D. Kumar, S. Seal, R. Kalyanaraman

**Affiliations:** 1 Bredesen Center, University of Tennessee, Knoxville, Tennessee 37996, USA; 2 Advanced Materials Processing and Analysis Center (AMPAC), NanoScience Technology Center (NSTC), Materials Science and Engineering (MSE) Department, Orlando, Florida 32816, USA; 3 NSF Engineering Research Center for Revolutionizing Metallic Biomaterials, North Carolina A&T State University, Greensboro, North Carolina 27411, USA; 4 Department of Material Science and Engineering, University of Tennessee, Knoxville, Tennessee 37996, USA; 5 Department of Chemical and Biomolecular Engineering, University of Tennessee, Knoxville, Tennessee, 37996, USA

## Abstract

We report a class of amorphous thin film material comprising of transition (Fe) and Lanthanide metals (Dy and Tb) that show unique combination of functional properties. Films were deposited with different atomic weight ratio (R) of Fe to Lanthanide (Dy + Tb) using electron beam co-evaporation at room temperature. The films were found to be amorphous, with grazing incidence x-ray diffraction and x-ray photoelectron spectroscopy studies indicating that the films were largely oxidized with a majority of the metal being in higher oxidation states. Films with R = 0.6 were semiconducting with visible light transmission due to a direct optical band-gap (2.49 eV), had low resistivity and sheet resistance (7.15 × 10^−4^ Ω-cm and ~200 Ω/sq respectively), and showed room temperature ferromagnetism. A metal to semiconductor transition with composition (for R < 11.9) also correlated well with the absence of any metallic Fe^0^ oxidation state in the R = 0.6 case as well as a significantly higher fraction of oxidized Dy. The combination of amorphous microstructure and room temperature electronic and magnetic properties could lead to the use of the material in multiple applications, including as a transparent conductor, active material in thin film transistors for display devices, and in spin-dependent electronics.

Functional amorphous thin films are attractive materials because they offer a combination of synthesis and property features that could lead to low cost of devices. For instance, disordered amorphous microstructure can be synthesized at room temperatures by physical vapor deposition techniques and can have very uniform microstructure over large areas due to the absence of grains and grain boundaries^1^. Amorphous materials also offer a rich toolbox for scientists because of the inherent complexity in relating their physical behavior to the disordered local structure and chemistry. In the past decade, the research into amorphous thin films for electronic applications has gained significant momentum in order to address growing societal needs for low cost and high quality electronic displays that can be fabricated over large ~m^2^ areas. One of the primary reasons for this interest in electronically-functional amorphous material was the report in 2004^2^ that transistors containing amorphous oxide thin films can be fabricated at room temperature and that they show excellent device characteristics.

In 1996 it was postulated that in order to obtain materials that possess amorphous microstructure and simultaneously show a combination of high optical transparency, carrier mobility, and conductivity, one should combine two or more metallic oxides made from metals with cation state configuration given by (n − 1)d^10^ns^0^ electronic configuration^3^. The reasoning was that the conduction band, which is formed by the overlap of spherically symmetric s-orbitals of the nearby metal cations, will lead to highly mobile electrons due to the large band curvature and also be more tolerant to local disorder in bond length and bond orientation. Indeed, all currently known high mobility (>10 cm^2^/V-s) transparent amorphous oxide materials, such as In-Ga-Zn oxide (a-IGZO) and Zn-In-Sn oxide, are made by combining two or more oxides that have extremely high mobilities. This desirable property arises from the metal cations of the oxides having an oxidation state configuration given by (n − 1)d^10^ns^0^, i.e. these are oxides with an s-conduction band^3^,^4^,^5^. The original postulate by Hosono *et al*. has been supported by materials genome calculations exploring a large fraction of the periodic table^6^ and has also been verified experimentally by independent groups^7^,^8^,^9^. Another materials approach is being pursued, which is based on the behavior of correlated electron systems that yields crystalline transparent conductors^10^. However, whether this approach can yield transparent amorphous conductors with desirable properties is not yet known.

Recently it was reported that oxide thin films made at room temperature by pulsed laser deposition from the metallic alloy, Terfenol-D, with composition of Fe (65.7 atomic %):Tb (10.3%):Dy (24%), showed an exciting combination of high transparency, very high Hall mobility, and a thermally stable amorphous microstructure^11^. As the authors showed, this material had a hall mobility *μ*_*H*_ greater than 32 cm^2^/V-s, that is exceedingly high for an amorphous material (for instance amorphous Si has <1 cm^2^/V-s, while amorphous In-Ga-Zn-O (*α*-*IGZO*), which is presently being commercialized for use in electronic display devices, has *μ*_*H*_ ~ 10 cm^2^/V-s)^4^,^8^. The authors also pointed out that low resistivity of this material, ~10^−5^ Ω-cm, could not be achieved if one considered the material to be a combination of the individual metal oxides such as the sesquioxides of *Fe*_2_*O*_3_, *Tb*_2_*O*_3_ or *Dy*_2_*O*_3_, as these are all well-known to be insulators, with the latter two being candidates for high-k dielectric applications^12^,^13^. In addition, Fe, Tb, and Dy have common oxidation states of 3d^5^4s^0^, 4f^9^6s^0^, and 4f^10^6s^0^ respectively, and with their partially filled d- and or f-subshells do not fit the general requirements for conducting amorphous oxides stated in ref. 3. Therefore this discovery clearly points to an alternate path from the (n − 1)d^10^ns^0^ cation configuration approach for creating new transparent amorphous oxide conductors and semiconductors. It has been hypothesized that these properties are likely a result of the formation of a ternary oxide from the original ternary alloy. While there are many pressing directions emerging from this work, one of the key questions was if the observed properties were uniquely related to the composition of the original alloy and the subsequent oxide that forms.

In this report, we have explored the role of metal composition with the hypothesis that since Fe is the largest component of the metal in the original Terfenol-D alloy, its ratio with respect to the Lanthanides could shed important clues about this material. For this reason, we have prepared films with Fe to Lanthanide metal (Tb + Dy) atomic weight ratio R varying between 0.60 to ~21. As we report here, all the films investigated show an unexpectedly rich variety of properties. The films were synthesized at room temperature by an electron beam co-evaporation process in high vacuum and all their physical properties were measured following exposure to air ambient. We confirmed that all the films showed a spatially uniform chemical composition through energy dispersive x-ray spectroscopy (EDS). All the films had an amorphous microstructure, as evidenced by glancing incidence x-ray diffraction measurements (GIXRD). X-ray photoelectron spectroscopy (XPS) revealed that the metal cation (Me^n+^) to metal (Me^0+^) ratio increased with decreasing R, while valence band edge analysis suggested that for R ≥ 11.9, the films were metal-like while the film for R = 0.60 had a energy band-gap. These composition dependent findings were supported by the optical transmission characteristics, which showed low transmission for films with R ≥ 11.9, and large transmission due to a direct optical band-gap for R = 0.60. All the compositions studied also yielded room temperature magnetic hysteresis behavior measured by a physical property measurement system (PPMS). These results point to an interesting class of metal-metal oxide thin film material that can exhibit potentially useful physical behavior, including the previously unobserved combination of amorphous microstructure, room temperature magnetism, low resistivity and sheet resistance (7.15 × 10^−4^ Ω-cm and 200.48 Ω/sq. respectively), and semiconductor-like direct-gap optical transitions.

## Results

### Composition and microstructure

**Figure 1:**
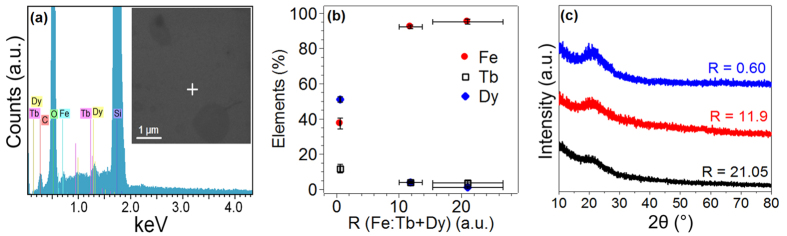
(**a**) Typical EDS point spectrum from the film with R = 0.60. Inset shows the SEM micrograph of the film and the plus sign marks the spot from where the point spectrum was obtained(**b**) Plot showing the atomic percentage of Fe, Tb and Dy in the three samples as a function of R (Fe: Tb + Dy) (**c**) GIXRD plots for typical films from the different R values.

The morphology and composition (as well as composition uniformity) for the different films were analyzed using scanning electron microscopy (SEM) and energy dispersive x-ray analysis in the SEM, respectively. Figure 1(a) inset shows the typical SEM micrograph of the films with R = 0.60. A featureless morphology reflecting the generally smooth films was evident; the dark spots present in the SEM image arises from ambient, organic contamination. The SEM images for the remaining two films can be found in the supplement. In Fig. 1(a) is the typical EDS point spectrum from a 57 nm × 57 nm sized area on the film. Quantitative analysis of the various peaks yielded the atomic % of each metal. The uniformity of the composition across the film was evaluated by performing similar EDS analysis at 5 random locations. We determined that all the films investigated were highly uniform in their spatial composition (refer to supplement for all EDS data), consistent with deposition by the e-beam evaporation process which normally yields good spatial uniformity. In Fig. 1(b), the cumulative results of the atomic % of the metallic elements present for the films prepared under the three different co-evaporation conditions is shown as a function of the atomic concentration ratio R defined as the atomic % of Fe to the atomic % of the Lanthanides (Tb + Dy). The primary change among the three films was in the concentration of Fe and Dy, while the concentration of Tb remained relatively unchanged. The three films had R of 0.60 ± 0.083, 11.9 ± 5.62 and 21.05 ± 5.62 respectively, and several films (up to 3 each) with these compositions with a fixed total thickness of 40 nm (calculated from pre-calibrated deposition rates of the targets in e-beam) were systematically investigated for various physical properties. In Fig. 1(c) the GIXRD scan from each of the compositions is shown. No evidence for crystalline scattering was evident and a broad peak centered around a 2*θ* of ~22° was observed in each case. Such featureless x-ray spectra with a single broad peak at low angles are generally evidence for lack of any long range order^14^,^15^ thus implying that all the film compositions deposited here were amorphous.

### Metal cation state

**Figure 2:**
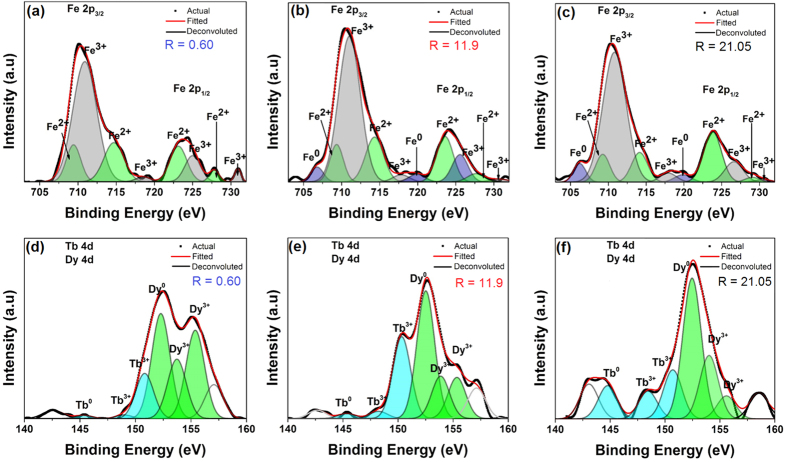
XPS plots showing (**a**–**c**) Fe 2p, and (**d**–**f**) Tb and Dy 4d for all three R values.

Knowing the oxidizing nature of the individual metals in this film, we next performed detailed investigations of the metal cation state (and oxygen content, which is presented in the supplement) in the films through XPS analysis. Figure 2(a–c) show the Fe 2p peaks for the three cases as well as deconvoluted spectrum obtained by peak fitting using Peakfit software (from Systat Software, Inc.). The deconvoluted peaks help identify the different cation states present. In films with R = 21.05 and R = 11.9, a peak at 706.2 eV and a satellite peak at 719.8 eV indicated the presence of Fe^0^, which is the metallic Fe; however for films with R = 0.60, these two peaks were missing. The predominant state of Fe in all three samples was Fe^3+^, with peaks centered at 710.82 eV, 711.09 eV, and 710.62 eV, and satellite peaks centered at 717.93 eV, 717.82 eV, and 718.54 eV for samples R = 21.05, 11.9 and 0.60 respectively. The samples also contained some Fe^2+^ that had peaks centered at 709 eV and satellite peaks centered at 714 eV for all three samples^16^. The relative concentration of each cation state was calculated using 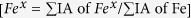, where *Fe*^*x*^ is the Fe atomic concentration of the cation state *x*, IA of Fe^x^ is the integrated area of the corresponding state *x*, and IA of *Fe* is the total integrated area of Fe peaks. Upon quantifying the percentage of the three Fe states from the XPS spectra, it was found that the percentage of Fe^0^, Fe^2+^ and Fe^3+^, respectively, were 8.21%, 28.12% and 63.67% for R = 21.05, 5.11%, 30.86% and 64.03% for R = 11.9, and 0%, 35.13% and 64.87% for R = 0.60. These values are tabulated in Table 1.

**Figure 3:**
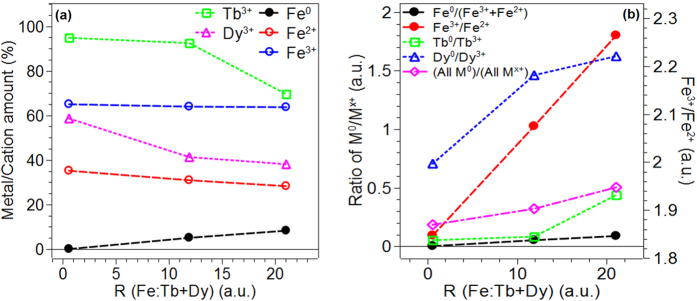
Plots of (**a**) metal and cation relative concentrations and (**b**) the ratio of metal to their cation states (left y-axis, black, green, magenta, and blue data points), and the ratio of Fe^3+^ to Fe^2+^ (right y-axis, red closed circles) as a function of R (Fe:Tb + Dy).

A similar analysis was performed for the Lanthanide metals. In Fig. 2(d–f)), the 4d peaks for both Tb and Dy are presented. The Tb^0^ peak was centered around 145 eV for all three samples, while the two peaks observed for Tb^3+^ were centered around 148 eV and 150 eV for the various compositions. Similar results were observed for Dy where the Dy^0^ peak was centered around 152 eV and Dy^3+^ peaks were centered around 154 eV and 155 eV. The deconvoluted Tb and Dy peaks were also quantified. The relative concentration of Tb^0^ and Tb^3+^ for each of the samples is shown in Table 1. For the case of Tb, as composition changed from R = 0.60 to R = 21.05, the concentration of metal Tb, i.e. Tb^0^ increased with concomitant decrease in that of Tb^3+^. A similar behavior was observed for the Dy, with the relative concentrations of Dy^0^ increasing and Dy^3+^ decreasing with increasing ratio R. Figure 3(a) demonstrates the trend in the relative concentration of Fe metal and of all the cations as R increases. The relative concentrations of all the metal cation percentages decrease with increasing value of R, and remarkably there is no metallic Fe for R = 0.60. Figure 3(b, right y-axis) shows the ratio of Fe^3+^ to Fe^2+^ as a function of R as well, and it shows almost a linearly increasing trend with increasing R. More importantly, for R = 0.60, the value of the ratio is 1.84, which has further significance as discussed later in the paper. In Fig. 3(b, left y-axis) the total metal (*M*^0^ = *Fe*^0^ + *Dy*^0^ + *Tb*^0^) to metal cation (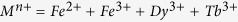) ratio is shown as a function of the composition ratio R (open circular symbols). From this it was evident that for compositions 0.60 and 11.9 more than 75% (84.5% and 76.3% respectively) of the metal was in oxidized state, while for the R = 21.05 case 66.5% was oxidized.

### Band-gap analysis

**Figure 4:**
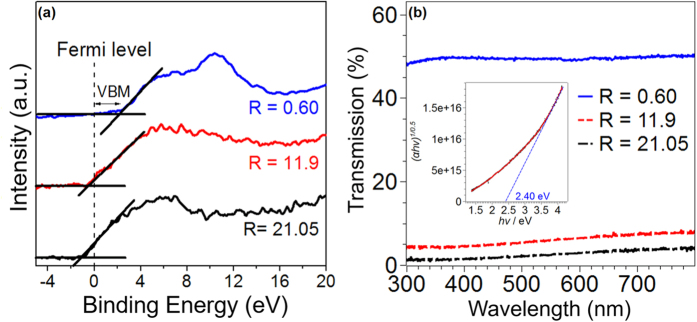
(**a**) XPS spectra for R = 0.60 (blue), 11.9 (red), and 21.05 (black) showing the valence band maximum. (**b**) Plot of percentage transmission vs the probing wavelength for all three samples; inset shows the Tauc plot for R = 0.60 generated from its transmission data using m = 1/2 for calculating a direct band-gap.

XPS spectra can provide very useful information on the position of the valence band edge with respect to the Fermi level, which, in turn, could help correlate the composition with physical properties. In Fig. 4(a) the valence band edge analysis was performed for all three compositions to ascertain the location of the valence band maximum (VBM) with respect to the position of the Fermi level (which corresponds to 0 eV binding energy on Fig. 4(a)). From the figure, it can be seen the VBM was quite different for the various compositions. The R = 11.9 and 21.05 cases had a VBM at −0.6 eV and −1.2 eV respectively, implying that the Fermi level was lower than the VBM, a behavior generally observed in metallic systems. On the other hand, the R = 0.60 composition had a VBM at 2.2 eV, implying that the VBM was below the Fermi level, a scenario typically seen in insulating or semiconducting material. To further establish the validity of these XPS findings, the broadband optical transmission spectra for the various compositions was analyzed and is shown in Fig. 4(b). The compositions of R = 11.9 and 21.05 had an extremely low transmission over the wavelength range investigated (which included the visible optical range between 300 and 800 nm), while the R = 0.60 case showed a significantly higher transmission of >45% throughout the range. This transparency measurement generally supported the XPS analysis of Fig. 4(a), i.e. the transmission of R = 11.9 and 21.05 were more consistent with a metallic system while R = 0.60 was more consistent with a semiconductor. To establish the nature and magnitude of the band-gap in R = 0.60, the Tauc plot was generated, and is shown in the inset of Fig. 4(b). From this plot, the direct band-gap of ~ 2.4 eV was estimated from the intercept, as shown on the figure. Therefore, by combining the measurement from the XPS (VBM is at 2.2 eV) and the optical band-gap value, the conduction band energy was ~0.2 eV above the Fermi level, implying that R = 0.60 should behave like an n-type semiconductor.

### Electrical properties

**Figure 5:**
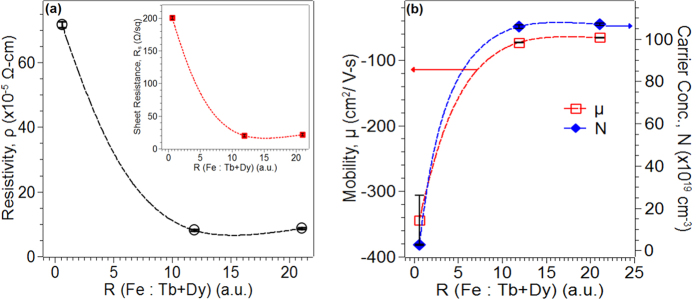
Plot of (**a**) four-probe resistivity (black, filled circles) with inset showing four-probe sheet resistance (red, filled rectangles), and (**b**) hall mobility (empty, red squares), and carrier concentration (filled, green diamonds; right y-axis) as a function of R (Fe:Tb + Dy).

To further understand (and differentiate) the various compositions, the room temperature electrical properties were measured using four probe technique. Figure 5(a) shows the resistivity *ρ* of the films, while the inset shows the sheet resistance Rs, as a function of composition ratio R. From this it was evident that a dramatic decrease in resistivity (and sheet resistance) occurred in going from R = 0.60 (*ρ* = 7.15 × 10^−4^ Ω-cm) to R = 11.9, and subsequently the resistivity was relatively unchanged (*ρ* *~* 9 × 10^5^ Ω-cm). This resistivity behavior was generally consistent with the results of XPS and optical transmission suggesting that R = 11.9 and 21.05 were likely metallic (low resistivity) while R = 0.60 was semiconducting (higher resistivity). In fact, the resistivity for the optically transparent film (having R = 0.60) is comparable to the reported resistivity for amorphous ITO films of similar thickness (5.93 × 10^−5^ Ω-cm for a 97 nm thick film)^17^. The resistivity could be further related to some fundamental quantities of the material through *ρ* = *1/μ*_*H*_*ne*, where *μ*_*H*_ is the Hall mobility, *n* is the carrier concentration and *e* is the electron charge. An independent measurement of the Hall voltage was used to extract the carrier concentration (with knowledge of the *ρ*) and the type of charge carriers (i.e. n- vs p-type). In Fig. 5(b), the measured Hall mobility is shown (open symbols) as a function of the composition. The sign of the mobility was negative (−*ve*) for all compositions, implying n-type carriers, and the mobility value for the semiconductor film (R = 0.6) was >300 cm^2^/V-s, while those for the two metallic films were 73 cm^2^/V-s (R = 11.9) and 65 cm^2^/V-s (R = 21.05). This finding was not surprising since metals are known to have lower hall mobility values than semiconductors^18^ From the measured *ρ* and *μ*_*H*_ values, the carrier concentration was estimated, and, as shown in Fig. 5(b, closed symbols) the carrier concentration dropped by approximately two orders of magnitude from the R = 11.9 and 21.05 compositions (*n* ~ 10^21^ cm^−3^) to the R = 0.60 case (*n* ~ 10^19^ cm^−3^), again supporting the semiconducting nature of R = 0.60.

### Magnetic properties

**Figure 6:**
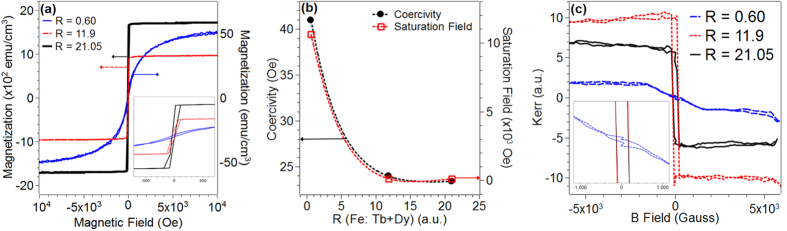
(**a**) Magnetization measurements at 300 K are plotted for all the compositions (left y-axis: black and red data; right y-axis: blue data); inset shows a magnified view near the zero field to highlight the coercivity in each of the plots. (**b**) Plot of the coercivity (left y-axis) and saturation field (right y-axis) as a function of composition for all the samples (**c**) Plot of Kerr rotation for all three compositions using magnetic field of −6000 Gauss to 6000 Gauss. Inset of plot shows a magnified view of the plots near zero field to highlight the coercivity.

Given the high concentration of iron in these films, we also measured the magnetic response as a function of composition. Figure 6(a) compares the room temperature magnetization of the three compositions measured using a PPMS instrument. All three compositions showed clear evidence for a saturation field as well as evidence for hysteresis with a small coercive field suggesting soft ferromagnetic behavior at room temperature. Also notable is the increase in the magnetization values as R values increased. In Fig. 6(b), the saturation field and the coercivity values are shown as a function of composition. Both quantities were found to increase in going from the metallic compositions (R = 11.9, 21.05) to the semiconducting state (R = 0.60). Upon calculating magnetization values for all the films, it was found that the values increased as R increased: 14.36 emu/cm^3^, 928.57 emu/cm^3^, and 1700 emu/cm^3^ for R = 0.60, 11.9 and 21.05 respectively. Furthermore, since the films showed magnetic moment all the way till 350 K, we concluded that the Curie temperature for films with all the R values investigated were ≥350 K. We also measured the the magnetic behavior using the Surface Magneto-Optical Kerr Effect (SMOKE) technique that detects change in incident light polarization with applied magnetic field. Shown in Fig. 6(c) are the relative Kerr intensities as a function of magnetic field measured at room temperature for the various compositions. These measurements also displayed hysteresis behavior with a saturation and coercivity similar to that measured using the PPMS [Fig. 6(a)] supporting the general observation that all the compositions investigated displayed soft ferromagnetism at room temperature.

## Discussion

From the XPS results, it was evident that the vacuum deposited metallic alloy film, for all the compositions investigated, was rapidly being oxidized upon exposure to air ambient at room temperature. This result was consistent with the recently reported observations that the the Terfenol-D composition (which has R ~ 1.94) also rapidly oxidizes upon air exposure to yield semiconducting films. From Fig. 3(c), it was evident that the oxidized metal made up ~50% or more of the total metal atoms and so therefore one could call these films a highly oxidized film. In this context, the transition from the metal to semiconducting behavior with composition can be correlated with certain important changes. First, we analyzed the Fe^3+/^Fe^2+^ ratio, and this was found to decrease from 2.26 in R = 21.05 to1.84 in R = 0.60, as demonstrated in Fig. 3(b). The value of 1.84 in R = 0.60 is very close to the value typically reported in magnetite (Fe_3_O_4_)^19^. On the other hand, the binding energy of Fe^3+^ of 710.82 eV and 711.09 eV in samples with R = 21.05 and 11.9 respectively can be assigned to the Fe_2_p_3/2_ value observed in *γ*-Fe_2_O_3_ (maghemite). Therefore, one important change in the film with decreasing R appears to be the change in the type of iron oxide, i.e. from predominantly *γ*-Fe_2_O_3_ to Fe_3_O_4_ (along with the simultaneous decrease in the amount of Fe^0^ content in the film). However, neither of these forms of iron oxide are known to have the combination of low resistivity and high mobility values measured here. Fe_3_O_4_ is reported to have resistivity of the order of 10^3^ Ω-cm^20^,^21^ while it is of the order of 10^3^ Ω-cm for *γ*-Fe_2_O_3_^22^. A 41 nm thickness epitaxial magnetite film has been reported to have an effective hall mobility of about 0.07 cm^2^/V-s with a maximum anomalous hall mobility of 35 cm^2^/V-s^23^. Although a hall mobility for a maghemite thin film was not available, the two oxides (magnetite and maghemite) are reported to have similar and low mobility values due to the narrowness of the d-bands^24^. Furthermore, there is little known about the electronic properties of these oxides in their amorphous state. In this context, it is tempting to assign the general behavior of these compositions as well as the transition from metallic to semiconducting state to the behavior of the Lanthanide elements. With reference to Fig. 3(a,b), it was evident that the metal cations of both the Lanthanide metals increased with decreasing R value. However, the Dy showed a dramatic change in the fraction of metallic to oxidized state, i.e. *Dy*^0^/*Dy*^3+^, dropped from >1.5 for R ≥ 11.9, to ~0.7 for R = 0.60. Such a large drop was not seen in any of the other metal constituents. Since this change in the Dy cation composition was strongly correlated with the measured optical and electronic changes, we hypothesize that the amongst other possible factors, the oxidation of the Dy metal is one of the most important drivers for the appearance of the novel properties being evidenced in these materials.

## Summary and Conclusion

In this study, thin films with varying composition atomic ratio of Fe:(Dy + Tb) (atomic percentages) were fabricated at room temperature. The films were found to be homogeneous in composition and had amorphous microstructure. Chemical analysis by XPS studies showed that while the metal cation fraction increased with decreasing atomic ratio Fe:(Dy + Tb), all the films had a large fraction of oxidized metal (≥66%). Furthermore, XPS valence band edge analysis suggested that high iron compositions were metallic while the high Lanthanide composition was semiconducting. These properties were supported by optical transmission and electrical resistivity measurements of the films. All the compositions were found to have ferromagnetism at room temperature. The finding of the composition-tunable metallic-semiconducting behavior, as well as the presence of room temperature ferromagnetism, optical transparency, and semiconducting behavior suggests the following: the combination of Fe with the Lanthanides (Dy and Tb) can lead to a class of materials with a range of multifunctional applications in the amorphous phase.

## Experimental Section

### Materials Synthesis

Quartz and thermally grown SiO_2_ on Si substrates were used for this study. The substrates were cleaned by first sonicating in 511-solution (1 part ammonium hydroxide, 1 part hydrogen peroxide, and 5 parts DI by volume) for 20 minutes, and then sonicating in DI for 20 minutes. They were then dried in air. Thin films (about 40 nm) of Fe:Tb:Dy were deposited using e-beam evaporation (Quad EV evaporator) inside a vacuum chamber at a base pressure of 5 × 10^−8^ Torr. The metal cation ratios were changed by co-depositing the film from two separate target crucibles: one containing pure iron metal (99.999%, Alfa Aesar), and the other containing a metallic alloy of iron (66%), terbium (10%) and dysprosium (24%) (commercially known as Terfenol-D, Etrema Products). Three different metal compositions were achieved by varying the deposition rate of the iron target with respect to that of Terfenol-D such that the deposition rates of iron to Terfenol-D were 4:1 (R = 21.05), 2:1 (R = 11.9), and 0:1 (R = 0.60). Following deposition, the samples were removed from the chamber, exposed to ambient air, and stored in metallic sample boxes under ambient conditions. For electrical measurements, silver (Ag) contact pads (~40 nm) were deposited on the four corners of some of the samples by first masking the samples appropriately with aluminum foil and then evaporating the Ag by e-beam at a base pressure of 2 × 10^−8^ Torr.

### Optical properties

Ultra-violet-Visible (UV-Vis) transmission spectra were obtained for the samples made on quartz using an Ocean Optics spectrometer with a He-Ne light source that allowed measurements to be made between 300 nm to 900 nm, with integration time of 1 ms and 100 scans to average. The beam spot size had a diameter of 1 mm. Transmission spectra were acquired at five different locations of each sample to ensure homogeneity of the film thickness. Tauc plot for composition R = 0.60 was generated by first converting the transmission values (% T) to absorbance using Beer-Lambert’s law and then dividing by the film thickness to get the absorption coefficient as a function of the probing wavelength. The Tauc plot was made with y-axis as (*αhv*)^1/m^ as a function of *hv* (the wavelength in energy units). A tangent was drawn at the region of the plot with sharp increase, which was then extrapolated to cut the x-axis at the band-gap value; m = 1/2 was used to obtain a direct band gap value.

### Material characterization

#### XPS

X-ray photoelectron spectrometer (XPS) measurement was accomplished at room temperature in an ion-pumped chamber (evacuated to 2 × 10^−9^ Torr) of an PE-PHI5400 spectrometer, employing Al-Kα radiation (BE = 1486.6 eV) of about 4 mm spot size. The binding energy (BE) for the samples was calibrated by setting the measured BE of C 1 s to 284.6 eV. Peakfit software was further used to identify the chemical state of multifaceted Fe 2p, Dy Tb 4d and O1s spectra according to the previous reports.

#### XRD

As-deposited films of all three compositions were characterized using grazing incidence XRD (GIXRD). The measurements were performed using a PANalytical Empyrean XRD system with a 1.8 KW Copper X-ray tube (1.54059 A). The samples were mounted on a motorized stage with Z-adjustment. A combination of beam mask and divergence slits were selected to illuminate the sample surface without illuminating the sample holder at a fixed incident angle. The diffracted x-rays were collected using a parallel plate collimator of 0.19 fixed divergence on the diffracted beam side. The GIXRD patterns were collected in the 2 theta range between 10–80° with a step size of 0.02° and step time of 1 sec/step.

#### EDX

Energy dispersive X-ray spectroscopy was performed on all three samples to quantify the bulk elemental metal ratios. The measurements were done using Merlin Zeiss SEM at 18 K magnification and EHT of 15 kV. The EDX spectra were deconvoluted and quantified using Esprit software by Bruker to obtain the atomic percentages of Fe, Tb, and Dy.

### Magnetic properties

The magnetic properties of the films made on SiO_2_/Si were studied using surface magneto-optical Kerr effect (SMOKE) technique in reflection mode. The SMOKE measurements were done in the longitudinal orientation using an s-polarized laser beam of 633 nm wavelength making 12.6 angle of incidence with the normal to the substrate plane, and a Si-diode detector. The signal was recorded at room temperature at frequencies of 100 kHz and 50 kHz for Kerr and ellipticity respectively. The applied magnetic field strength ranged from −6000 Gauss to 6000 Gauss. 50 scans were taken for averaging and removing background noise. PPMS measurements were also performed on the samples to measure the magnetoresistance at different temperatures. The magnetic properties of the three samples were measured using vibrating samples magnetometer. The room temperature magnetization was measured by maintaining the sample at 300 K in presence of magnetic field (100, 200 or 500 Oe). The coercivity of each sample was measured by recording magnetization versus field loops with applied fields of up to 2T applied parallel to the plane of the film.

### Electrical properties

Electrical measurements were made on the samples with Ag contact pads. Van der Pauw sheet resistance and hall measurements were made following the NIST technique described in ref. 11 with the samples mounted on an Ecopia SPCB-1 board that allows contacts to be made via spring loaded gold pins.

## Additional Information

**How to cite this article**: Taz, H. *et al*. Transparent ferromagnetic and semiconducting behavior in Fe-Dy-Tb based amorphous oxide films. *Sci. Rep.* **6**, 27869; doi: 10.1038/srep27869 (2016).

## Supplementary Material

Supplementary Information

## Figures and Tables

**Table 1:** Cation composition of the various metals measured by XPS for the three composition ratios investigated.

Cation	Composition ratio		
	*R* = 0.60	11.9	21.05
Fe^0^	0	5.11	8.21
Fe^2+^	35.13	30.86	28.12
Fe^3+^	64.87	64.03	63.67
Tb^0^	5.09	7.48	30.52
Tb^3+^	94.91	92.52	69.48
Dy^0^	41.38	60.40	61.87
Dy^3+^	58.61	41.38	38.13
