# Supplementary material for: Transparent ferromagnetic and semiconducting behavior in Fe-Dy-Tb based amorphous oxide films
Source: Sci Rep. 2016 Jun 14;6:27869. doi: 10.1038/srep27869 (PMC4906280; doi:10.1038/srep27869)
Supplement: Supplementary Information [file srep27869-s1.pdf]

Supplementary Information for "Transparent ferromagnetic and semiconducting behavior in Fe-Dy-Tb based amorphous oxide films"

Humaira Taz,[†] Tamilselvan Sakthivel,[‡] Nana K. Yamoah,[¶] Connor Carr,[§] Dhananjay Kumar,[‖] Sudipta Seal,[‡] and Ramki Kalyanaraman[*,§,⊥,†]

†*Bredesen Center, Knoxville, TN*

‡*Advanced Materials Processing and Analysis Center (AMPAC), NanoScience Technology Center (NSTC), Materials Science and Engineering (MSE) Department, Orlando, FL*

¶*Engineering Research Center, Greensboro, NC*

§*Department of Materials Science and Engineering, Knoxville, TN*

‖*Department of Mechanical Engineering,Greensboro, NC*

⊥*Department of Chemical and Biomolecular Engineering, Knoxville, TN*

E-mail: ramki@utk.edu

Composition and Microstructure

For films with all the three R values, SEM images were obtained to show a featureless film, as evident in the insets of the plots in Fig. 1(a,b,c). EDS point spectra were obtained from 5 locations (each $57nm \times 57nm$), which were subsequently quantified to obtain atomic percentages of Fe, Tb and Dy. Fig. 1(a,b,c) show the atomic percentages from all five locations for films with R = 0.60, 11.9 and 21 respectively, confirming a homogeneous distribution of

the three elements.

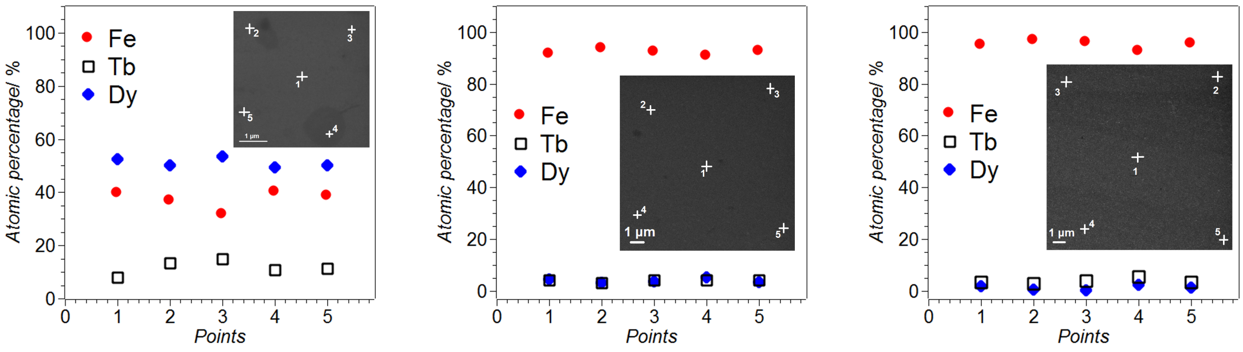

(a) (b) (c)

Figure 1: Plots showing quantified atomic percentages of Fe, Tb and Dy from the five EDS point spectra for (a) R = 0.60, (b) R = 11.9, and (c) R = 21. The inset in each plot shows the SEM image of the film with the respective R value, with regions marked by white plus signs (numbered) to show the locations where the EDS spectra were obtained from.

Metal Cation State

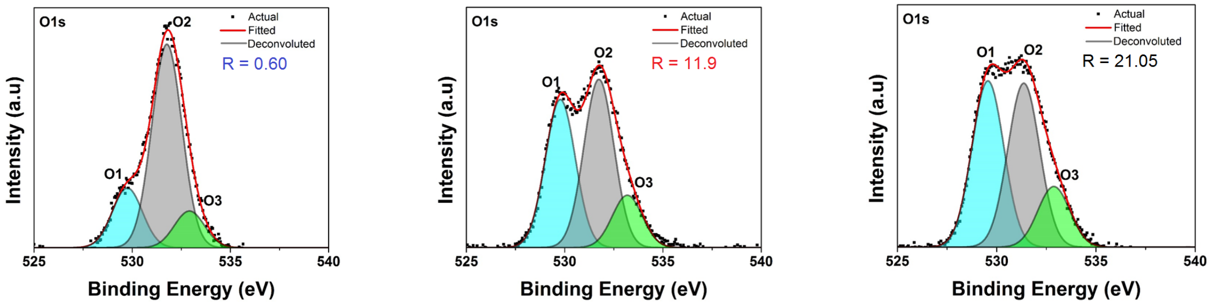

(a) (b) (c)

Figure 2: XPS plots (a), (b), (c) showing O1s spectra for all three R values.

The XPS peaks corresponding to oxygen in such oxide systems, i.e. O1s peaks, are generally known to be broad with multiple overlapping components[1,2]. In 2(c), the O1 peak centered at 529.6 eV was seen in all three compositions and is generally attributed to the Fe metal oxide, but, according to literature, can also correspond to the oxides from Tb and Dy.[1,3] The O2 peak marked and centered around 531.7eV may be attributed to the OH-

species that are mostly present on the surface. The formation of the hydroxide could be due to the reduction of the oxygen in the air to hydroxide at the metal surface by the moisture in the air. The metal is thus oxidized to cations and the corresponding metal hydroxide also forms very rapidly. The high binding energy peak centered at around 533eV belongs to the adsorbed moisture on the surface. According to literature, this peak also belongs to SiO_2; however it is not a plausible explanation in our case because the deposited film thickness is about 40nm. Overall, the integrated area of the O1 and the O2 peaks was seen to increase with the decreasing R value, thus supporting the changing metal oxide content with composition ratio R.

References

(1) Castle, J. E. *The Journal of Adhesion* **2008**, *84*, 368–388.

(2) Liu, G.; Liu, A.; Zhu, H.; Shin, B.; Fortunato, E.; Martins, R.; Wang, Y.; Shan, F. *Advanced Functional Materials* **2015**, *25*, 2564–2572.

(3) Nemoshkalenko, V.; Uvarov, V.; Borisenko, S. *Journal of Electron Spectroscopy and Related Phenomena* **1995**, *76*, 641 – 646, Proceedings of the Sixth International Conference on Electron Spectroscopy.
